# Examining Whether Offspring Psychopathology Influences Illness Course in Mothers With Recurrent Depression Using a High-Risk Longitudinal Sample

**DOI:** 10.1037/abn0000080

**Published:** 2016-02

**Authors:** Ruth Sellers, Gemma Hammerton, Gordon T. Harold, Liam Mahedy, Robert Potter, Kate Langley, Ajay Thapar, Frances Rice, Anita Thapar, Stephan Collishaw

**Affiliations:** 1 Institute of Psychological Medicine and Clinical Neurosciences, School of Medicine, MRC Centre for Neuropsychiatric Genetics and Genomics, Cardiff University, and School of Psychology, Andrew and Virginia Rudd Centre for Adoption Research and Practice, University of Sussex; 2 Institute of Psychological Medicine and Clinical Neurosciences, School of Medicine, MRC Centre for Neuropsychiatric Genetics and Genomics, Cardiff University; 3 School of Psychology, Andrew and Virginia Rudd Centre for Adoption Research and Practice, University of Sussex, and Institute of Genetic Neurobiological and Social Foundations of Child Development, Tomsk State University; 4 Institute of Psychological Medicine and Clinical Neurosciences, School of Medicine, MRC Centre for Neuropsychiatric Genetics and Genomics, Cardiff University; 5 School of Psychology, Cardiff University; 6 Institute of Psychological Medicine and Clinical Neurosciences, School of Medicine, MRC Centre for Neuropsychiatric Genetics and Genomics, Cardiff University; 7 Department of Clinical, Educational, and Health Psychology, University College London; 8 Institute of Psychological Medicine and Clinical Neurosciences, School of Medicine, MRC Centre for Neuropsychiatric Genetics and Genomics, Cardiff University

**Keywords:** maternal depression, offspring depression symptoms, offspring disruptive behavior, psychopathology, direction of effects

## Abstract

Depression is known to be influenced by psychosocial stressors. For mothers with recurrent depressive illness, the presence of psychopathology in their children may have important effects on their own mental health. Although the impact of maternal depression on child mental health is well-established, no study to date, as far as we are aware, has examined the extent to which offspring psychopathology influences the course of depression in mothers with a history of recurrent depressive illness, what types of child psychopathology impact maternal mental health, or whether risks vary by child gender. Aims were to (a) Use a longitudinal design to examine whether adolescent psychopathology (depression, disruptive behavior disorder; DBD) predicts recurrence of a depressive episode and depression symptom *course* in women with a history of recurrent depression; and (b) To test if observed effects vary by child gender. 299 mothers with recurrent major depressive disorder and their adolescent offspring were assessed on 2 occasions, 29 months apart. Maternal depression and offspring psychopathology were assessed using semistructured interview measures. Cross-generational links across time were assessed using structural equation modeling. Analyses were adjusted for past severity of maternal depression. Offspring depression symptoms but not DBD symptoms at baseline predicted future episode recurrence in mothers. Depression symptoms in daughters (β = .16, *p* = .039) but not sons (β = −.07, *p* = .461), predicted an increase in maternal depression symptoms across time. Psychopathology in daughters is associated with long-term depressive symptoms in women (mothers) with a history of recurrent depression. Findings highlight the importance of careful assessment and management of mental health problems in adolescents for more effective management of maternal depression. This study suggests that offspring symptoms of depression may be important for the recurrence of maternal depression episodes. Girls’ symptoms of depression may be a particularly important psychosocial stressor for the development of depressive symptoms in mothers with a history of recurrent depression.

## Abstract (general public)

This study suggests that offspring symptoms of depression may be important for the recurrence of maternal depression episodes. Girls’ symptoms of depression may be a particularly important psychosocial stressor for the development of depressive symptoms in mothers with a history of recurrent depression.

Adult depression is common, is associated with significant impairment, and is now the second leading cause of global disability (Ferrari et al., 2013). Depression often shows a chronic or recurrent illness course (Blazer, Kessler, McGonagle, & Swartz, 1994), with prior episodes of depression being one of the best predictors of future depression (Burcusa & Iacono, 2007). However, there is also considerable heterogeneity in illness course in adults with depression, and given the individual and public health burden of depression, it is important to understand what contributes to more or less favorable outcomes.

Depression illness factors such as severity, number of prior episodes, age at onset, and adult psychiatric comorbidity are all important predictors of future recurrence (Lewinsohn, Rohde, Seeley, Klein, & Gotlib, 2000; Mueller et al., 1999; Solomon et al., 2000), but other (potentially modifiable) psychosocial risk mechanisms are less well understood. Comparisons of depressed and nondepressed adults highlight elevated rates of recent negative life events and chronic psychosocial stress (Kendler, Gardner, & Prescott, 2002), and these have also been shown to predict depression onset (Gilman, Kawachi, Fitzmaurice, & Buka, 2003; Kessler & Magee, 1993; Pine, Cohen, Johnson, & Brook, 2002). However, the contribution of psychosocial stress to depression relapse and illness course is mixed (Mazure, Bruce, Maciejewski, & Jacobs, 2000; Monroe, Slavich, & Gotlib, 2014; Spinhoven et al., 2011).

Exposure to psychopathology in offspring is a potentially important psychosocial stressor. As far as we are aware, no study has tested the extent to which exposure to psychopathology in offspring predicts depression illness course in mothers with recurrent depression. This is an important issue for several reasons. First, women of child-rearing age are a high-risk group for depression (Kessler, 2003). Second, family adversity has been highlighted as an important proximal risk factor for depression (Kendler et al., 2002). Third, rates of family stress often increase when becoming a parent, particularly when children develop emotional or behavioral problems (Feske et al., 2001; Raposa, Hammen, & Brennan, 2011). Finally, depression can be characterized by increased sensitivity to adverse circumstances, therefore mothers with a history of depressive illness may be especially vulnerable to the stressful effects of psychopathology in their children. Understanding the extent to which adolescent psychopathology contributes to the maintenance or worsening of parental depression has potentially important implications for clinical management of adult depression: it may provide an important indication of whether successful prevention and intervention for offspring may also have benefits for depressed mothers.

Previous research has shown that children’s psychopathology might impact on parent distress and depression symptom levels in general population samples (Allen, Manning, & Meyer, 2010; Guo & Slesnick, 2011; Kouros & Garber, 2010; Nicholson, Deboeck, Farris, Boker, & Borkowski, 2011). Very little is known, however, about the extent to which child psychopathology impacts on depression recurrence and symptom course in mothers with a history of depression. The impact of offspring psychopathology on parent depression illness course may be particularly important to examine during adolescence given increasing rates of depression and disruptive behavior problems during this time (Costello, Copeland, & Angold, 2011; Rutter, 2007). One recent study of adolescents diagnosed with clinical depression found that trajectories of adolescent and maternal depressive symptoms were intercorrelated, especially for mothers reporting at least one depressive symptom at baseline (Perloe, Esposito-Smythers, Curby, & Renshaw, 2014). This suggests that adolescent and maternal depressive symptoms may change together, especially where mothers have already experienced symptoms of depression. However, this study did not specifically examine the direction of the association between maternal depression and adolescent symptoms. Another community-based study provides additional evidence that adolescent psychopathology is associated with concurrent and future episodes of maternal depression (Feske et al., 2001), but again did not explicitly test the direction of effects underlying these associations or the impact on symptom course in mothers with a history of recurrent depression.

It is necessary to consider that depression can be viewed as a dimensional construct (Hankin, Fraley, Lahey, & Waldman, 2005; Kendler & Gardner, 1998), and symptom severity is an important guide to risk for future episodes and levels of functional impairment (Angold, Costello, Farmer, Burns, & Erkanli, 1999; Fergusson, Horwood, Ridder, & Beautrais, 2005; Pickles et al., 2001). Therefore, it is important to examine the extent to which potential risk factors are associated with both new episodes of depression (recurrence), as well as with maternal depression symptoms across time (depression symptom course).

The role of child gender also merits attention in relation to adult-offspring psychopathology interplay. Psychopathology increases across adolescence, with rates differing by gender. First, from puberty onward, adolescent girls experience higher rates of depression compared with boys (Essau, Conradt, & Petermann, 2000; Ge, Lorenz, Conger, Elder, & Simons, 1994; Hankin et al., 1998; Lewinsohn, Hops, Roberts, Seeley, & Andrews, 1993; Thapar, Collishaw, Pine, & Thapar, 2012). Disruptive behaviors also increase during adolescence (Rutter, 2007), with boys being more likely to be aggressive and antisocial than girls (Lewinsohn et al., 1993; Maughan, Rowe, Messer, Goodman, & Meltzer, 2004; McDermott, 1996). Furthermore, the magnitude of association between maternal depression and offspring psychopathology has been found to differ for sons and daughters in some studies (Cortes, Fleming, Catalano, & Brown, 2006; Guo & Slesnick, 2011; Jaffee & Poulton, 2006; Lewis, Rice, Harold, Collishaw, & Thapar, 2011). Second, evidence suggests that the relationship with parents may differ by child gender. The nature and intensity of mother–son and mother–daughter interactions varies in important ways (Raley & Bianchi, 2006). For example, evidence from population cohorts shows that parental concern, parental monitoring, and time spent with parents are higher for adolescent girls than adolescent boys (Collishaw, Gardner, Maughan, Scott, & Pickles, 2012). Therefore, it is possible that mothers experience daughters’ psychopathology as more concerning and more stressful, and that influences on maternal depression may therefore be greater for girls. Where child effects on parents have been tested separately by gender, some evidence suggests that symptomatology in girls, but not boys, may be associated with increased risk for maternal depression symptoms (Guo & Slesnick, 2011; Jaffee & Poulton, 2006), but not all evidence is consistent (Leung et al., 2009). Evidence concerning possible gender differences in effects on symptom course in mothers with depression is lacking. Research is therefore needed to clarify whether adolescent psychopathology impacts on the course of maternal depression differently for male and female offspring.

The objective of the present study was to utilize a prospective longitudinal study to examine whether adolescent offspring symptoms of depression and disruptive behavior disorder (DBD) predicted variation in depression course among mothers with a history of recurrent depressive disorder. Specific aims were (a) to examine the role of offspring psychopathology (symptoms of depression and DBD) in prediction of a recurrence of maternal depressive disorder over the course of the study, (b) to use a structural equation modeling approach to test transactional effects between adolescent and maternal symptoms across time, and (c) to examine whether results varied according to child gender. In order to focus on proximal influences, analyses were adjusted for past maternal depression severity. We hypothesized that adolescent symptomatology would predict greater risk of future maternal depression, and that associations between psychopathology in offspring and later maternal depression would be stronger for girls than boys. To reduce biases associated with shared-rater effects, we utilized a cross-rater approach: mothers reported on their depression symptoms and offspring reported on their own depression and DBD symptomatology.

## Materials and Methods

### Sample

This study used data from the Early Prediction of Adolescent Depression (EPAD) study, a high-risk study of the offspring of recurrently depressed parents. The sample and procedure have been described in detail elsewhere (Mars et al., 2012; Sellers et al., 2013).

Parents were recruited predominantly from primary care (general practice surgeries) in South Wales, United Kingdom, on the basis of treatment for at least two episodes of *Diagnostic and Statistical Manual of Mental Disorders*, 4th edition (*DSM–IV*) major depressive disorder (confirmed at interview). Children were aged 9 to 17 years old at first assessment. One child from each family was selected to participate in the study. If there was more than one child in the age range, the youngest child was selected to avoid bias in parental selection of children.

The sample at baseline consisted of 337 parents with recurrent depression (315 mothers, 22 fathers), and their offspring (197 females, 140 males; age 9–17 years, mean 12.4 years). Two families were excluded due to a diagnosis of bipolar disorder in the affected parent after the first assessment. Analyses were conducted with mothers only (*n* = 313). Mothers were aged 26–55 (mean 41.2 years). Fourteen families were excluded as the child was not living at home throughout the course of the study (eligible *n* = 299).

Longitudinal data were necessary to examine the direction of the associations between maternal depression symptom levels and offspring psychopathology. We examined the direction of effects using data from the first assessment and at follow-up, which was conducted 29 months (*SD* 3.6) after the initial assessment.

At baseline, 23% of the children were identified as having a psychiatric disorder including depressive disorders (major depressive disorder, dysthymia, and depression not otherwise specified, NOS), anxiety disorder (generalized anxiety disorder, separation anxiety, social phobia, panic disorder, agoraphobia, and obsessive–compulsive disorder), DBDs (oppositional defiant disorder, conduct disorder, and disruptive behavior NOS), attention-deficit/hyperactivity disorder (ADHD), eating disorder, and bipolar spectrum disorder.

### Procedure

Data were collected from parents and children via semistructured diagnostic interviews. The study was approved by Multicenter Research Ethics Committee. Families were interviewed at their home (99%) or at the University Hospital of Wales. Written informed consent or assent was obtained from all participants as appropriate. Further information regarding the procedures is detailed elsewhere (Mars et al., 2012).

### Measures

Child symptoms of major depressive disorder (MDD) and DBDs. Child symptoms of MDD and DBD were assessed using a semistructured interview, the Child and Adolescent Psychiatric Assessment (Angold & Costello, 2000). Child reports were used to examine the presence of symptoms of depression and DBD (conduct disorder/oppositional defiant disorder) in the preceding 3 months. The total number of child *DSM–IV* symptoms of depression and DBD, at both baseline and at follow-up, were summed to create depression and DBD symptom counts at both time points. Descriptive data on child symptoms and rates of disorder at baseline and follow-up are presented in Table 1. Interrater reliability was high (average κ = .94).[Table-anchor tbl1]

Maternal depression symptom levels. The number of maternal *DSM–IV* depression symptoms was assessed using the Schedules for Clinical Assessment in Neuropsychiatry (SCAN; Wing et al., 1990), a semistructured clinical interview assessing depression symptoms in the preceding month. Symptom counts ranged from 0–9. Descriptives of maternal symptoms at baseline and follow-up are presented in Table 1.

Between the baseline and follow-up assessment, additional information was collected regarding maternal *DSM–IV* MDD using the SCAN and a life-history calendar approach (Belli, 1998; Caspi et al., 1996). The presence of *DSM–IV* MDD occurring between the baseline assessment and follow-up was established using this data to derive a measure of MDD recurrence. Depression recurrence was considered as a binary variable; where mothers reported MDD at any point between the baseline assessment and follow-up, recurrent depression was considered to be present.

### Preliminary Analyses and Missing Data

All analyses adjusted for past maternal depression severity. Interviews at baseline ascertained parents’ periods of hospitalization for depression and impairment of the worst two episodes of depression using Global Assessment of Functioning (GAF) scores (American Psychological Association, 1994). Past maternal depression severity was defined as a GAF score <30 or any hospitalization due to depression in accordance with previous criteria (Hammen & Brennan, 2003). Furthermore, this measure of maternal severity has previously been shown to be associated with negative child outcomes in the current sample (Mars et al., 2012). Past maternal severity was coded as a dichotomous variable (yes/no), with 28.6% reporting at least one severe episode of depression. There was weak evidence of an association between maternal past depression severity and baseline maternal depression symptoms (boy model: *b* = .31, 95% CI [−.01, .62], *p* = .057; girl model: *b* = .26, 95% CI [−.01, .53], *p* = .057). There was evidence of an association between maternal past depression severity and baseline adolescent depression symptoms for girls (boy model: *b* = .14, 95% CI [−.07, .36], *p* = .197; girl model: *b* = .22, 95% CI [.01, .43], *p* = .041), but not between maternal past depression severity and baseline adolescent DBD symptoms (boy model: *b* = −.08, 95% CI [−.36, .20], *p* = .561; girl model: *b* = .19, 95% CI [−.03, .40], *p* = .085).

Given the large age range of the children in the current study (age 9–17 years at baseline), the current study also adjusted for child age. Child age at baseline was associated with girls’ symptoms of depression (girls *b* = .08, 95% CI [.04, .13], *p* = .001; boys *b* = .02, 95% CI [−.03, .07], *p* = .369). Child age at baseline was also associated with child symptoms of DBD (girls *b* = .12, 95% CI [.08, .16], *p* = .001; boys *b* = .08, 95% CI [.01, .15], *p* = .018). Both past maternal depression severity and child age were therefore included in the models, but not presented for ease of interpretation.

Of the eligible sample (*n* = 299), 14 families refused to participate at follow-up (4.7%). Of the remaining sample (*n* = 285), 72 (*n* = 25%) had partially complete data (36 families completed questionnaire assessments only and therefore did not have interview data. An additional 36 families had incomplete interview assessments, and therefore, symptom counts could not be computed). Therefore, complete data at baseline and follow-up was available for 73% (*n* = 213/299) of the sample.

Families who had missing data at follow-up included mothers with higher depression symptoms at baseline (missing at follow-up *M* = 3.44, *SD* = 2.74; participated at follow-up *M* = 2.38, *SD* = 2.58, *p* = .004). There was weak evidence of an association between missing data at follow-up and offspring depression symptoms (missing at follow-up *M* = 1.48, *SD* = 1.87; participated at follow-up *M* = 1.04, *SD* = 1.51, *p* = .057). Child DBD symptoms at baseline were not associated with missingness at follow-up (missing at follow-up *M* = 3.47, *SD* = 2.41; participated at follow-up *M* = 2.90, *SD* = 2.41, *p* = .522). Missing data at follow-up was also found to vary by maternal education (*p* = <.001) and child IQ (*p* = <.001). To account for observed patterns of selective attrition, missing data for offspring symptomatology, maternal depression, and other covariates were imputed using multivariate imputation by chained equations (Van Buuren & Oudshoom, 2000) using all available data. This assumes that data are missing at random (MAR) that is, given the observed data included in the imputation model, the missingness mechanism does not depend on the unobserved data (White, Royston, & Wood, 2011). The variables associated with nonresponse were therefore included in the imputation model to make the assumption of MAR as plausible as possible, along with other measures closely associated with maternal and offspring psychopathology (child emotional and conduct symptoms and maternal depression symptoms assessed using questionnaire screening measures at multiple time points) and all other variables included in analyses (White et al., 2011).

Imputation models were run using binary and ordinal logistic and linear regression models as appropriate. Predictive mean matching (PMM) was used when continuous variables were not normally distributed. PMM yields acceptable estimates while maintaining the underlying distribution of the data (Vink, Frank, Pannekoek, & van Buuren, 2014). Where gender interactions were tested, imputation models were run by child gender. All variables with missing data used in analyses were imputed up to the maximum sample size of *n* = 299. Thirty imputed datasets were derived each with 10 cycles of regression switching and then all analyses were run on imputed datasets by combining estimates using Rubin’s rules (White et al., 2011). All analyses presented show the results using the imputed sample of 299. Complete case analyses were also conducted and are shown in supplementary material.

### Analysis

Initial binary logistic regression analyses examined the associations between child depression and DBD symptoms at baseline and the presence of maternal depressive episode recurrence after baseline (i.e., throughout the duration of the study period yes/no). Unadjusted models, and analyses controlling for past maternal depression severity and household income, are reported using risk ratios and 95% confidence intervals. Analyses were conducted in STATA version 13 (StataCorp, 2007).

Cross-lagged and reciprocal effects models utilize longitudinal data to test the direction of effects between variables (Kline, 2005). The cross-lagged model involved simultaneously estimating the contribution of each variable at baseline (maternal depression symptoms and child psychopathology) in accounting for each of the variables at follow-up (maternal depression symptoms and child psychopathology) while controlling for the previous correlation between the two constructs at baseline and the stability in the constructs over time. Reciprocal effects models are used to test the direction of effects that exist within time rather than across time, again controlling for the stability of each variable across time.

Cross-lagged and reciprocal effects models tested links between child symptomatology (depression and DBD) and maternal depression symptom levels (all continuous measures). Subgroup comparisons using stacked modeling procedures (Bollen, 1989) were used to assess whether the magnitude of parameter estimates differed in strength for boys and girls. Post hoc Wald tests were used to test the assumption of equality between the targeted paths (effect of child symptomatology on maternal depression symptoms) across gender. A significant Wald test statistic provides an estimate of the statistical significance of any specific parameter comparisons (i.e., whether there are any differences in effect by child gender).

Past maternal depression severity was included as a covariate in all analyses. Note that in structural equation modelling (SEM) analyses, covarying for differences in the constructs at the first measurement occasion accounts for the influence of the covariate at the later time point via the various indirect pathways (Little, Preacher, Selig, & Card, 2007). To test whether results were influenced by covarying patterns of social disadvantage, sensitivity analyses further adjusted for household income.

Structural equation model analyses were undertaken in Mplus 7.11 (Muthén & Muthén, 1998) using robust maximum likelihood estimation procedures.

## Results

### Maternal Depressive Recurrence

Sixty-three percent of mothers reported at least one episode of depression between the baseline and follow-up period. After adjusting for child age, there was evidence of an association between child depression symptoms at baseline and maternal depressive episode at follow-up (RR = 1.14, 95% CI [1.04, 1.24], *p* = .003), which weakened when adjusting for past maternal depression severity and family income, falling below conventional levels of significance (RR = 1.09, 95% CI [1.00, 1.19], *p* = .064). Results show that there was almost a 10% increase in risk of mothers having a recurrent episode of *DSM–IV* depressive disorder per increase in each child depression symptom. There was little evidence for an association between child DBD symptoms at baseline and maternal depressive episode at follow-up in unadjusted (RR = 1.08, 95% CI [.98, 1.19], *p* = .103) or adjusted models (RR = 1.06, 95% CI [.97, 1.17], *p* = .211).

### Cross-Lagged Panel Analyses

Correlations for all study variables are presented in Table 2.[Table-anchor tbl2]

### Offspring Depression

#### Boys

**Figure 1 fig1:**
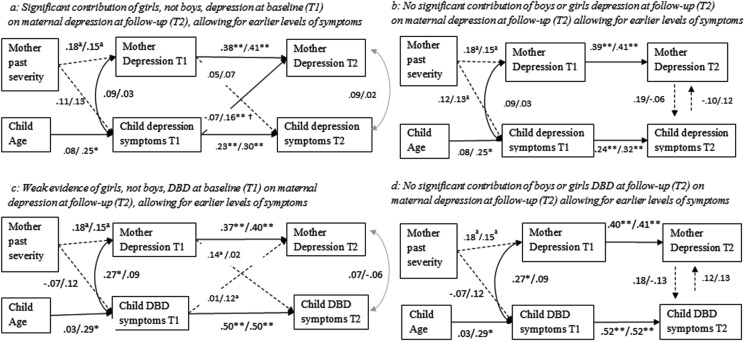
The relationship between offspring depression symptoms and maternal depression symptom course across (panel a) and within (panel b) time, and between offspring disruptive behavior disorder (DBD) symptoms and maternal depression symptom course across (panel c) and within (panel d) time. Coefficients for boys are presented first, followed by girls. Cross-lagged model saturated; no goodness of fit statistics generated. T1 = Time 1; T2 = Time 2. ^†^ Significant difference between two pathways. ^a^ *p* < .10. * *p* < .05. ** *p* < .01.

Figure 1a shows results for the cross-lagged model linking boys’ depression symptoms and maternal depression levels after adjusting for maternal past depression severity and child age. The stability coefficients for maternal depression levels and boys’ depression symptomatology were moderate (β = .38, 95% CI [.20, .56], *p* < .001; β = .23, 95% CI [.04, .41], *p* < .001, respectively) and were not significantly different from each other. No longitudinal cross-lagged effects were observed between boys’ depression symptoms (baseline) and maternal depression levels at follow-up (β = −.07, 95% CI [−.30, .11], *p* = .461), or between maternal depression levels (baseline) and boys’ depression levels at follow-up (β = .05, 95% CI [−.13, .22], *p* = .589). Tests of reciprocal effects models (Figure 1b) demonstrated no evidence of influences of boys’ depression symptoms on maternal depression symptom levels (β = −.10, 95% CI [−.41, .27], *p* = .617), or vice versa (β = .19, 95% CI [−.22, .60], *p* = .928).[Fig-anchor fig1]

#### Girls

Figure 1a shows results for the cross-lagged model linking girls’ depression symptoms and maternal depression symptom levels after adjusting for maternal past depression severity and child age. The stability coefficients across time for maternal depression and girls’ depression symptoms were moderate (β = .41, 95% CI [.28, .54], *p* < .001; β = .30, 95% CI [.14, .46], *p* < .001, respectively) and did not significantly differ from each other. There was evidence of a moderate effect of girls’ depression symptoms at baseline on maternal depression symptoms at follow-up (β = .16, 95% CI [.03, .40], *p* = .039), but not between maternal depression levels (baseline) and girls’ depression symptoms at follow-up (β = .07, 95% CI [−.09, .23], *p* = .376), suggesting that girls’ depression symptoms were associated with an increase in maternal depression symptom levels across time, but not vice versa. Tests of reciprocal effects models (Figure 1b) demonstrated no evidence of influences of girls’ depression symptoms on maternal depression symptom levels (β = .12, 95% CI [−.25, .48], *p* = .602), or vice versa (β = −.06, 95% CI [−.45, .32], *p* = .748).

The Wald test examined differences between male and female offspring by comparing specific parameters between boys and girls. The path from child depression symptoms (baseline) to maternal depression symptoms (follow-up) differed for boys and girls (Wald (1) = 3.74, *p* = .042).

Analyses were also conducted using complete case analysis and are presented in supplemental Figure 1 (panels a and b). The pattern of results was similar; however, the effect of girls’ depression symptoms on maternal depression was slightly weaker (β = .14, 95% CI [−.03, .30], *p* = .108).

### Offspring Disruptive Behaviors

Figure 1c shows cross-lagged models for offspring DBD symptoms and maternal depression symptoms. No longitudinal cross-lagged effects were observed between boys’ DBD symptoms and maternal depression symptoms. There was weak evidence of an effect of girls’ DBD symptoms on maternal depression symptoms, but this association fell below conventional levels of significance (β = .12, 95% CI [−.03, .26], *p* = .096). Tests of reciprocal effects models (Figure 1d) demonstrated no evidence of influences of boys’ or girls’ DBD symptoms on maternal depression symptom levels, or vice versa.

Analyses were also conducted using complete case analysis and are presented in Figure 1 (panel c and d). The pattern of results was the same.

Sensitivity analyses also examined household income as an additional potential confounder. Approximate gross family income was assessed by parent report questionnaire at baseline. Household income was coded on a scale of 1 (<£10,000) to 7 (>£60,000), with lower scores indicating lower household income (see Table 3). Income was associated with maternal depression symptoms (boy model: β = −.10, 95% CI [−.18, −.02], *p* = .015; girl model: β = −.05, 95% CI [−.12, .01], *p* = .090) but not child symptoms of depression (boys: β = −.03, 95% CI [−.09, .02], *p* = .291; girls: β = −.04, 95% CI [−.09, .01], *p* = .141) or DBD (boys: β = −.04, 95% CI [−.12, .02], *p* = .224; girls: β = −.02, 95% CI [−.07, .03], *p* = .497). Sensitivity analyses additionally covaried for household income in cross-lagged models. The pattern of results remained unchanged with girls’ symptoms of depression at baseline remaining associated with maternal depression symptoms at follow-up (β = .17, 95% CI [.01, .35], *p* = .050) and weak evidence of effects of girls’ DBD symptoms at baseline on maternal depression symptoms at follow-up (β = .14, 95% CI [−.02, .30], *p* = .083).[Table-anchor tbl3]

## Discussion

A number of important studies have highlighted risk to offspring from having a parent with depression (Beardslee, Versage, & Gladstone, 1998; Brennan et al., 2000; Weissman, Warner, Wickramaratne, Moreau, & Olfson, 1997). The current study took a different approach by examining the extent to which child psychopathology impacts on maternal depressive symptom course and illness recurrence. Findings indicate that in mothers with a previous history of depressive disorder, offspring depression symptoms during adolescence predict an exacerbation of depressive illness in mothers. Mothers with a past history of recurrent depression experienced a 9% increase in risk for a further episode of depression at follow-up for each additional offspring symptom of depression reported at study baseline after accounting for background risk (household income and past maternal depression severity). Structural equation models provided further evidence of risk effects of offspring depression on the course of maternal depression, particularly so in the case of mother–daughter dyads. Furthermore, this association was not due to shared rater variance as children reported on their own symptoms. Findings for effects of DBD on maternal depression symptom course were inconclusive, with only weak evidence of an association between girls’ DBD symptoms (at baseline) and maternal depression symptoms at follow-up.

It is well-established that maternal depression is associated with increased risk for offspring psychopathology when compared with children of nondepressed mothers (Goodman & Gotlib, 1999), and the rate of offspring psychiatric disorder in this sample was high (24%) when compared with general population rates (∼11%; Green, McGinnity, Meltzer, Ford, & Goodman, 2004). In keeping with previous findings from this sample (Mars et al., 2012), maternal past depression severity was also associated with variation in offspring depression symptoms within this high-risk group.

It is important to examine whether child psychiatric symptoms have an adverse effect on the course of maternal depression to better understand risk factors that underlie the maintenance or recurrence of adult depression. We extended previous research by examining this in a sample of mothers with a history of recurrent episodes of major depression. Findings showed that offspring symptoms of depression (but not DBD) predicted a future episode recurrence in mothers, even when accounting for prior maternal depression severity. Cross-lagged and reciprocal effects models provided further and more stringent tests of possible transactional effects between child and maternal psychopathology over time by simultaneously taking into account associations between variables at baseline and follow up, and considering the stabilities of both maternal depression and child symptomatology. Therefore, although the main focus of the study was to examine child effects on maternal mental health, the modeling allows for baseline maternal depression symptom levels to predict change in child outcomes. Furthermore, by examining the association between child and maternal symptoms (using a cross-lagged approach), we also examined whether child gender moderated observed associations. Girls’ symptoms of depression predicted an increase in maternal depression levels over time using cross-lagged models, and effects for girls were greater than for boys. Findings were not explained by cross-sectional correlation of maternal and child symptoms at baseline, by previous maternal depression severity, or by variation in household income.

Findings of gender differences are in accord with a growing body of literature suggesting that maternal depression may be more strongly associated with girls’, rather than with boys’, depression (Davies & Windle, 1997; Fergusson, Horwood, & Lynskey, 1995). Previous studies have suggested that girls’ symptoms of depression and DBD predict maternal depression symptoms in the general population (Guo & Slesnick, 2011; Jaffee & Poulton, 2006), although findings of so-called child effects on parent have been mixed. We focused on adult patients who had been treated in primary care. For this group, our findings suggest that the presence and impact of daughters’ depression symptoms on mothers is important to consider when examining risk factors that contribute to the course of depression in women during offspring adolescence.

Although the focus of this study was to test child effects on parents, it is important to note that, in contrast to some previous research (Allen et al., 2010; Hughes & Gullone, 2010; Kouros & Garber, 2010; Nicholson et al., 2011), we did not observe bidirectional longitudinal effects between maternal depression levels and later offspring DBD or depression. This is not to suggest that maternal depression as a risk for offspring psychopathology is not important; the study was not designed to test this, as control participants were not included. This is because maternal depression is a well-established risk factor for offspring psychopathology (Brennan, Hammen, Katz, & Le Brocque, 2002; Lieb, Isensee, Höfler, Pfister, & Wittchen, 2002; Wickramaratne & Weissman, 1998). One possible explanation for why we did not observe effects of maternal depression symptom levels on change in offspring psychopathology is that the current study utilized a high-risk sample of parents, all of whom had a long history of recurrent depressive illness, and other aspects of maternal depression may be more important in terms of predicting variation in risk for the development of psychopathology within such high-risk samples. For example, severity, chronicity and recurrence of maternal depression episodes have been found to impact on risk for offspring psychopathology in this and other high-risk samples (Hammen & Brennan, 2003; Mars et al., 2012). Because of all offspring being exposed to some level of maternal depression, it may be less likely (than in case-control studies) that fluctuations in maternal depression levels at any specific time point would influence change in child symptomatology. Moreover, the time frame in which symptom change was examined was fairly long, on average 29 months, and this may have influenced results. In addition, it is important to note that more substantial variation in maternal depression symptoms evident for example in treatment trials (Weissman et al., 2006) may also show a more pronounced impact on change in offspring psychopathology over time.

### Limitations and Future Directions

Findings should be considered in light of study limitations. First, sample size limitations meant it was not possible to test for potential age-group differences. We adjusted for child age in the main analyses, but future research should examine whether the effect of daughters’ mental health problems on maternal depression course differs across different developmental periods (Gross, Shaw, Moilanen, Dishion, & Wilson, 2008; Jaffee & Poulton, 2006; Pilowsky et al., 2008). Second, there were too few fathers with recurrent depression participating in the current study to examine influences of offspring psychopathology on the course of paternal depression. Research suggests that paternal depression is an important risk factor for the development of offspring psychopathology (Brennan et al., 2002; Elgar, Mills, McGrath, Waschbusch, & Brownridge, 2007; Ramchandani, O’Connor et al., 2008; Ramchandani, Stein et al., 2008), but there is little research examining whether child psychopathology has adverse influences on the course and maintenance of paternal depression (Ge, Conger, Lorenz, Shanahan, & Glen, 1995; Gross et al., 2008). Future research should also clarify the relationship between parent and child psychopathology in father–son and father–daughter dyads. Third, as in most longitudinal studies, there was some evidence of selective attrition; for example, mothers with greater symptom levels at baseline were less likely to participate at follow-up. We used multiple imputation to reduce attrition biases, and conducted sensitivity analyses with similar findings across imputed and complete case analyses. One important difference was that, in complete case analyses, the path from girls’ depression symptoms to maternal depression symptoms was slightly weaker compared with analyses using multiple imputation, and wider confidence intervals meant that the association fell below conventional levels of significance. However, it is important to consider that any difference in the effect size (β = .16 in imputed analyses vs. β = .14 in complete cases) was minimal. Furthermore, in complete cases, the association between offspring depression symptoms and maternal depression symptoms may be underestimated because where both the mother and the child had elevated depression symptoms, families were more likely to have missing data. In addition, by including variables that predict missingness in the imputation, multiple imputation should help correct for biases that may be present in complete case analyses (Sterne et al., 2009). Reported findings using multiple imputation may therefore present more reliable estimates of longitudinal associations in this sample. Fifth, although the focus of the current study was to investigate child “effects” on the course of maternal depression, the design is observational, and we cannot assume associations across time are necessarily causal. Although a strength of the study is the longitudinal design, we cannot rule out the contribution of other time-varying factors impacting on both mother and child and contributing to changes in symptom levels in both.

It was beyond the scope of the current study to test mechanisms that might explain observed risk associations, but previous studies provide a number of possible explanations. Psychopathology in offspring is likely an important source of stress for parents (Crnic & Low, 2002), perhaps because children become harder to manage, create parental and external concerns, and impact on parental self-esteem. Psychopathology in offspring may lead to difficulties in parenting or family relationships, such as increased parent–child conflict, interparental conflict, or family discord (Branje, Hale, Frijns, & Meeus, 2010; Combs-Ronto, Olson, Lunkenheimer, & Sameroff, 2009; Gross et al., 2008; Hale et al., 2011). As depression is characterized by increased sensitivity to adverse circumstances, mothers with a history of depression are likely to be at greater risk than nondepressed mothers to the effects of offspring psychopathology (Jaffee & Poulton, 2006). The differential patterns by child gender imply that these factors are especially relevant for girls and their mothers.

Evidence from general population samples suggests that mothers demonstrate more parental concern, monitoring, and spend more time with adolescent daughters than adolescent sons (Collishaw et al., 2012). It is therefore plausible that mothers find daughters’ psychopathology more stressful and concerning. In addition, there is an increase in exposure to stressful life events around adolescence, and adolescent girls are thought to be more sensitive to the depressogenic effects of exposure to stressful life events in the context of parent depression (Bouma, Ormel, Verhulst, & Oldehinkel, 2008). It could be that some of the stressful life events that adolescent girls are exposed to are also stressful for their mothers and thus impact on depressive symptoms in both mothers and daughters. Finally, earlier onset of depression in girls may be a marker of greater familial liability for depression. While adjustment for past maternal depression severity did not explain the effect of girls’ mental health problems on the course of maternal illness, other aspects of shared liability may be important to consider.

### Clinical Implications

The findings of the current study suggest that for clinicians who deal with adult depression, asking about offspring psychopathology is important and relevant to the assessment and ongoing management of depression, particularly given the relatively high risk of depression in women of child-rearing age. In addition, improved communication and coordination between different services dealing with adult depression and children’s mental health may assist with more effective monitoring and management of mental health problems in affected families. In particular, there are a range of nonhealth care agencies providing care to children and adolescents with mental health problems (such as social services, youth offending services, and education), and often little information from these agencies is available to clinicians dealing with adult mental health problems. Effective prevention and treatment of offspring psychopathology is an important priority in its own right, and findings from this study highlight that there may be additional benefits for the management of adult depression.

## Supplementary Material

10.1037/abn0000080.supp

## Figures and Tables

**Table 1 tbl1:** Mean Symptom Scores and Rates of Disorder for Boys and Girls and Maternal Depression at Baseline and Follow-Up Using Complete Cases

	Boys		Girls	
	Baseline	Follow-up	Baseline	Follow-up
Mean DBD symptoms (*SD*)	1.79 (2.10)	1.84 (2.17)	1.73 (1.66)	1.56 (1.84)
Range	0–9	0–9	0–6	0–11
Mean depression symptoms (*SD*)	.94 (1.28)	1.11 (1.37)	1.27 (1.78)	1.55 (2.09)
Range	0–8	0–7	0–9	0–9
Any disorder, % (*n*)	24.2 (30)	24.5 (26)	22.3 (39)	23.6 (34)
DBD,^a^ % (*n*)	8.1 (10)	12.3 (13)	5.7 (10)	5.6 (8)
Mood disorder,^b^ % (*n*)	2.4 (3)	6.6 (7)	6.9 (12)	13.2 (19)
	Maternal depression			
	Baseline	Follow-up	Baseline	Follow-up
Mean depression symptoms (*SD*)	2.39 (2.60)	1.53 (2.12)	2.76 (2.67)	2.17 (2.67)
Major depressive disorder recurrence, % (*n*)	—	57.3% (55)	—	63.8% (86)
^a^ DBD: disruptive behavior disorder (oppositional defiant disorder or conduct disorder). ^b^ Mood disorder: major depressive disorder, dysthymia, bipolar, cyclothymia.				

**Table 2 tbl2:** Correlations Between Clinical Measures at Both Time Points

	1	2	3	4	5	6
1. Time 1 maternal depression symptoms	—	.10	.11	.42**	.10	.21*
2. Time 1 child depression symptoms	.06	—	.34**	−.05	.26**	.10
3. Time 1 child disruptive behavior symptoms	.03	.37**	—	.01	.19*	.45**
4. Time 2 maternal depression symptoms	.49**	.25**	.25**	—	.09	.16*
5. Time 2 child depression symptoms	.08	.35**	.30**	.25**	—	.27**
6. Time 2 child disruptive behavior symptoms	.05	.26**	.53**	.13	.45**	—
*Note.* Top right of table shows correlations among study variables for boys. Bottom left shows correlations among study variables for girls.						
* *p* < .05. ** *p* < .001.						

**Table 3 tbl3:** Demographic Sample Description

	Mean (*SD*)/ Percentage (*n*)
Child gender (% male)	41.5% (124)
Child age at baseline	12.29 (1.98)
Income	
<£10,000	14.7% (40)
£10,000–£20,000	15.4% (42)
£20,000–£30,000	20.6% (56)
£30,000–£40,000	17.6% (48)
£40,000–£50,000	10.3% (28)
£50,000–£60,000	9.2% (25)
>£60,000	12.1% (33)
Single parent household	29.1% (87)
Education (no qualifications)	17.6% (48)
Past severity	28.6% (85)
