# Supplementary material for: Examining Whether Offspring Psychopathology Influences Illness Course in Mothers With Recurrent Depression Using a High-Risk Longitudinal Sample
Source: J Abnorm Psychol. 2016 Feb;125(2):256–66. doi: 10.1037/abn0000080 (PMC4745386; doi:10.1037/abn0000080)
Supplement: Supplementary file 1 [file abn-ABN-2014-0472-revision.docx]

Child depression symptoms T2

Mother Depression T2

(CAPA) T3

.47**/.44**

.26**/.32**

Child depression symptoms T1

.07/-.06

.14/.01

*Panel 1b: No significant contribution of boys or girls depression at follow-up (T2) on maternal depression at follow-up (T2) allowing for earlier levels of symptoms*

Mother Depression T2

(CAPA) T2

Child depression symptoms T1

Child depression symptoms T2

*Panel 1a: No significant contribution of boys depression at baseline (T1) on maternal depression at follow-up (T2), allowing for earlier levels of symptoms*

Mother Depression T1

.16^a^/.28*

.12/.19*

.17 / .28*

.12 / .19*

Mother past severity

Mother past severity

.10/.09

-.07/.13 †

.47** /.44**

Mother Depression T1

.07 / -.06

.28** / .32**

.25 /.02

-.11 /.03

Child Age

.14/.26*

Child Age

.14 / .26*

Child DBD symptoms T2

Mother Depression T2

(CAPA) T3

.46**/.45**

.42**/.40**

Child DBD symptoms T1

.19*/-.04

.15/.01

*Panel 1d: No significant contribution of boys or girls DBD at follow-up (T2) on maternal depression at follow-up (T2) allowing for earlier levels of symptoms*

Child DBD symptoms T1

Child DBD symptoms T2

*Panel 1c: No significant contribution of boys or girls DBD at baseline (T1) on maternal depression at follow-up (T2), allowing for earlier levels of symptoms*

Mother Depression T1

.17/.29*

-.04/.12

-.01/.12

.12 /.05

Mother past severity

.48**/.45**

-.07/.10

Mother Depression T2

(CAPA) T2

.42**/.41**

.24/-.06

.17/.29*

Mother Depression T1

-.04/.12

Mother past severity

Child Age

Child Age

.21*/.38*

.21*/.38*

.19*/-.04

*Supplementary figure 1: The relationship between offspring depression symptoms & maternal depression symptoms course across (panel a) & within (panel b) time, & between offspring DBD symptoms & maternal depression symptoms course across time (panel c) & within time (panel d). Coefficients for boys are presented first, followed by girls.*

Notes: ^a^ p<.10, * p<.05, **p<.01, T1 Time 1; T2 Time 2. Cross-lagged model saturated; no goodness of fit statistics generated; † significant difference between two pathways (†Wald test (1) =3.04, p.081).
